# The Impact of the COVID-19 Pandemic on Cancer Care and Health-Related Quality of Life of Non-Hispanic Black/African American, Hispanic/Latina and Non-Hispanic White Women Diagnosed with Breast Cancer in the U.S.: A Mixed-Methods Study Protocol

**DOI:** 10.3390/ijerph182413084

**Published:** 2021-12-11

**Authors:** Chiara Acquati, Tzuan A. Chen, Isabel Martinez Leal, Shahnjayla K. Connors, Arooba A. Haq, Anastasia Rogova, Stephanie Ramirez, Lorraine R. Reitzel, Lorna H. McNeill

**Affiliations:** 1 Graduate College of Social Work, University of Houston, 3511 Cullen Blvd, Houston, TX 77204, USA; 2 Department of Health Disparities Research, The UT MD Anderson Cancer Center, 1515 Holcombe Blvd, Houston, TX 77030, USA; lmcneill@mdanderson.org; 3 HEALTH Research Institute, University of Houston, 4849 Calhoun Road, Houston, TX 77204, USA; tchen3@central.uh.edu (T.A.C.); imarti31@central.uh.edu (I.M.L.); lrreitzel@uh.edu (L.R.R.); 4 Department of Psychological, Health and Learning Sciences, University of Houston, 491 Farish Hall, Houston, TX 77204, USA; connorss@uhd.edu (S.K.C.); aahaq2@central.uh.edu (A.A.H.); arogova@central.uh.edu (A.R.); 5 Department of Social Sciences, University of Houston-Downtown, Houston, TX 77002, USA; 6 College of Natural Sciences and Mathematics, University of Houston, 3507 Cullen Blvd, Houston, TX 77204, USA; sramirez5@uh.edu

**Keywords:** COVID-19, health disparities, health-related quality of life, cancer care, cancer care disruption, care transitions, breast cancer, women’s health, ethnic and racial minorities, mixed-methods design

## Abstract

The COVID-19 pandemic has had critical consequences for cancer care delivery, including altered treatment protocols and delayed services that may affect patients’ quality of life and long-term survival. Breast cancer patients from minoritized racial and ethnic groups already experience worse outcomes, which may have been exacerbated by treatment delays and social determinants of health (SDoH). This protocol details a mixed-methods study aimed at comparing cancer care disruption among a diverse sample of women (non-Hispanic White, non-Hispanic Black/African American, and Hispanic/Latina) and assessing how proximal, intermediate, and distal SDoH differentially contribute to care continuity and health-related quality of life. An embedded mixed-methods design will be implemented. Eligible participants will complete an online survey, followed by a semi-structured interview (with a subset of participants) to further understand factors that influence continuity of care, treatment decision-making, and self-reported engagement. The study will identify potentially modifiable factors to inform future models of care delivery and improve care transitions. These data will provide the necessary evidence to inform whether a subsequent, multilevel intervention is warranted to improve quality of care delivery in the COVID-19 aftermath. Additionally, results can be used to identify ways to leverage existing social resources to help manage and support patients’ outcomes.

## 1. Introduction

### 1.1. COVID-19 Is a Health Disparities Issue

The outbreak of Coronavirus Disease 2019 (COVID-19) has affected 230 million people worldwide, with 42 million cases registered in the USA and approximately 688,000 deaths as of summer 2021 [1,2]. COVID-19 disproportionately burdens minoritized racial and ethnic minorities [3], such as Black/African American and Hispanic/Latinx adults who have higher rates of COVID-19 infection than their non-Hispanic White (NHW) counterparts [3,4,5,6]. These minority groups are also overrepresented among hospitalized patients with the disease [7] and face proportionally higher mortality rates [4,8,9]. This is at least partially attributable to the exacerbation of pre-existing racial and health inequalities associated with social determinants of health (SDoH) [10,11,12], and rising rates of unemployment and loss of medical insurance [13,14,15] that have a greater effect on minority groups’ financial resources [16,17,18]. These same groups have lower incomes and higher proportions of poverty [19], public insurance or being uninsured [20,21] compared to NHWs. 

Racial and ethnic minority groups share the unequal burden of being more likely to be low-income essential workers [22] and unemployed [23,24], forcing these populations to make economic cutbacks during the pandemic [22], which induces elevated stress and anxiety [22]. Residential racial segregation has been also consistently linked with a variety of adverse health outcomes, underlying health conditions, and affects minoritized groups more than NHWs [25,26,27]. Access to health services is contingent upon insurance and user-pay systems, which may disproportionately negatively affect those from minority groups [28]. Additionally, these groups may not have the health literacy skills necessary to fully respond to pandemic messaging [29] or to evaluate true versus misinformation on the virus. Consequently, racial and ethnic minority groups are likely to experience reduced life expectancies that are three to four times larger than in NHWs during the COVID-19 pandemic [30]. In sum, COVID-19 disparities in incidence and mortality should be situated in the context of multiple factors that affect health-related outcomes, such as material resources deprivation, access to social networks, and chronic stress due to structural discrimination [12].

### 1.2. COVID-19 and Cancer Care Delivery

Cancer patients have an increased risk of contracting COVID-19 [7,31], being hospitalized [7,31], and succumbing to the virus [32]. This vulnerability is due to being immunocompromised as a result of the malignancy itself and systemic anticancer therapies, resulting in higher susceptibility to severe infections and complications [32,33,34,35,36]. Thus, their long-term survival may be particularly affected by delays in surgery and administration of anticancer therapies [33,34,35,36]. Breast cancer patients in active treatments, compared to other groups, are the most likely to be infected with COVID-19 [7]. Notably, Black/African American breast cancer survivors are more likely to be diagnosed with COVID-19 compared to their NHW counterparts; the largest racial disparity for infection compared to other cancers [7]. 

The pandemic has had critical consequences for cancer care delivery [33,34,35,36,37,38,39,40,41,42,43,44,45,46] including a reduction in cancer screenings [47], diagnoses [47,48], and surgeries [47]. Recommendations and guidelines for triage, prioritization and altered treatment regimens [49,50,51,52,53,54] have also contributed to modifications to care protocols and the transition to tele-medicine services [8,13,35,36,37,38,39,40,41,47,48]. However, this adaptation of treatment pathways can have long-term implications for the timely detection of disease progression and complications that may affect cancer outcomes [13,39,40,55]. In May 2020, 79% of cancer patients in active treatment experienced some delay in their health care because of COVID-19 [56]; which may affect long-term survival because of suboptimal or delayed care [8,13,39,40,41,55,57,58,59]. Together, these trends can exacerbate pre-existing disparities in cancer morbidity and mortality experiences by Black/African American and Hispanic/Latinx adults [47,60,61,62].

In terms of breast cancer care, the pandemic has led to reductions in screening/diagnostic mammography [47,63,64], diagnoses [47,48,65], surgeries [47,66] including breast reconstruction [66,67] and genetic counseling/testing [63,64]. The reduction in the number of biopsies (71%) and diagnoses (51.8%) is higher in breast cancer compared to other cancers [47,48]. Additionally, registered breast cancer treatment delays [64,65,68,69,70,71,72], may be due to fear of COVID-19 infections [66,69,70,71,72,73]. Disruptions to oncology services negatively impact emotional well-being, anxiety and depression experienced by breast cancer patients, which have been associated with greater emotional vulnerability and poor cognitive function [68,69]. Breast cancer patients from racial and ethnic minority groups already experience significantly more cancer treatment delay [74,75,76,77,78], which may exacerbate COVID-19 related treatment delays and psychosocial outcomes in these groups [68,77]. 

### 1.3. Examining Breast Cancer Care and Minority Women’s Outcomes in the Context of Social Determinants of Health Is Necessary to Fully Understand COVID-19’s Impact on Health Disparities 

The disproportionate burden of breast cancer is well documented, with Black/African American and Hispanic/Latina women reporting worse health outcomes than their NHW counterpart [79,80,81,82,83,84,85,86,87,88,89,90]. For instance, in Texas, where breast cancer is the leading cancer diagnosis, mortality is 44% higher for Black/African American than NHW women [79,91]. Disparities exist in incidence, mortality, and importantly, survivorship care [79,80,81,82,83,84,85,86,87,88,89,90]. Traditional explanations have included biologic differences in tumor characteristics [81], late-stage diagnosis due to lack of cancer screening [91,92,93], and socioeconomic factors [83,94]. However, a growing body of literature suggests that differences in selection and adherence to recommended treatments may play a major role in the maintenance of disparities in breast cancer outcomes [83,95,96,97,98,99,100,101,102,103,104,105,106,107,108,109]. In the current oncologic practice, where timely engagement with treatment is crucial to prevent recurrence and reduce mortality [89,90], differences in treatment initiation and adherence to treatment have become increasingly relevant given the present recommendations for triage and modified delivery of cancer care [75,95,96,97,98,99]. Black/African American women are four times more likely to experience treatment delays and less likely to receive cancer-directed surgery; additionally, Black/African American and Hispanic/Latina women also fail to receive definitive local therapy, chemotherapy, and radiotherapy [99,100,101]. Minority breast cancer patients are disproportionally characterized by non-initiation, discontinuation, and non-adherence to adjuvant endocrine therapy [98,99,100,101,102,103,104], which leads to lower survival, shorter time to recurrence, increased medical costs, and lower quality of life [87,98,99,100,101,102,103,104,105]. Key determinants of disparities range from proximal factors (socio-demographic variables), to intermediate (social network characteristics), and distal factors (access to resources; healthcare system characteristics, and policy) [88,89,90,92,93,94,95,96,97,98,99,100,101,102,103,104,105,106,107,108,109]. Although multiple levels of contextual influences affect behavior, currently available interventions to alleviate breast cancer disparities fail to comprehensively address these SDoH, or the interplay between them [106]. For minoritized racial and ethnic women receiving treatment for breast cancer, the effects of SDoH coupled with COVID-19 altered treatment protocols [54,110,111,112] may exacerbate the existing disproportionate burden of the disease [80,81,82,83,95,105,106]. More research is therefore needed to understand these potential effects and how to address them to achieve breast cancer health equity.

### 1.4. The Current Protocol

The present paper describes an NCI-funded mixed-methods study protocol investigating the impact the COVID-19 pandemic has on the receipt of optimal breast cancer care among a diverse sample of women (non-Hispanic Black/African American, Hispanic/Latina, and NHW). For the purpose of this investigation, groups were defined according to the classification from the U.S. Office of Management and Budget (OMB), which is used for the 2020 Census categorization of race and ethnicity. Individuals who identify with the ethnicity of Hispanic/Latinx may be of any race. This research project will fill a significant gap in current understanding of the long-term implications of the pandemic on cancer disparities by assessing differential rates of cancer care disruption and health-related quality of life among non-Hispanic Black/African American and Hispanic/Latina women (relative to non-Hispanic White women) diagnosed with early-stage breast cancer. The study will also examine how proximal, intermediate, and distal SDoH differentially contribute to these outcomes to inform future interventions able to sustain equitable models of care delivery. 

## 2. Materials and Methods

### 2.1. Study Design

The study utilizes an embedded mixed methods design [113], chosen for the purpose of complementarity, in which the addition of qualitative data is used to enhance or elaborate upon the results of quantitative analyses. Each component will be used to capture overlapping, but distinct aspects of participants’ experiences [113], i.e., quantitative data will include psychosocial outcome measures, while qualitative data will focus on capturing the context of care such as women’s personal experiences of barriers and processes in accessing care. In this embedded design, quantitative and qualitative data will be collected concurrently, in which the second qualitative strand builds on the first quantitative strand and the qualitative interview participants are selected from among the survey respondents, integrating strands on the methods level through connection via the sampling frame [114]. Integration will also occur during the interpretation and reporting stages where the analyses from the two strands are integrated and compared through use of a joint display table. Integration will be achieved through using qualitative findings to elaborate or enhance upon quantitative results, yielding a more comprehensive view of participants’ experiences and barriers to care.

Study procedures will occur in two virtual “visits.” In the first visit, eligible patients complete an online survey assessing proximal, intermediate, and distal factors affecting care continuity and quality of life. In the second visit, a semi-structured interview is conducted with a selection of self-referred women to assess factors that influenced their access to and continuity of care. 

Funding for the current protocol was obtained from the National Cancer Institute of the National Institute of Health (3P20CA221697-04S1), supported by grants P20CA221696 (to Lorna H. McNeill) and P20CA221697 (to Lorraine R. Reitzel).

#### 2.1.1. Conceptual Framework

**Figure 1:**
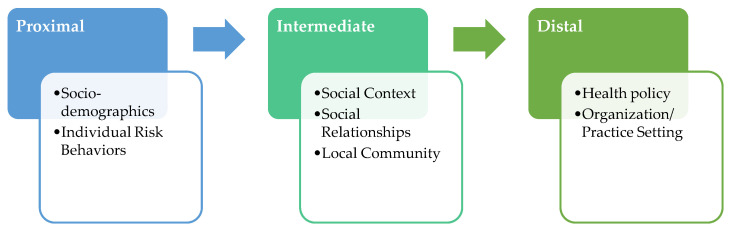
Conceptual model of the study.

The conceptual framework of the study is based on the National Institute on Minority Health and Health Disparities (NIMHD) Research Framework [115,116] and the Centers for Population Health and Health Disparities (CPHHDs) [117,118] multilevel model, which are grounded in socio-ecological theory [119]. As outlined in Figure 1, the study targets (1) proximal, (2) intermediate, and (3) distal determinants of health disparities to identify which of them differentially influences cancer care receipt, patient-reported outcomes, and the interplay between them. Distal determinants include policies and organization/practice settings that affect the availability, receipt of, and quality of health care [115,117]. Intermediate determinants include social context, physical environment, and social relationships [115,117,120,121]. Social relationships refer to social networks, which are forms of social capital that suppress the negative effects of impoverished social environments. These negative effects can, in the absence of such networks, be increased by social isolation [122,123,124,125,126,127,128]. The physical environment includes availability and accessibility of local health care resources; transportation, quality air and water, healthy food; presence of crime; and neighborhood characteristics [129,130]. Finally, proximal determinants are embedded in the individual and include socioeconomic status, race/ethnicity, gender identity, and cultural beliefs; they also include engagement in risk behaviors [115,116,118]. The conceptual model of the study is informed by the taxonomy proposed by Taplin and Rodgers [131] about factors influencing the quality of cancer care and ultimately cancer-related health outcomes. This model also integrates recent evidence-based recommendations for the development of multilevel interventions to address racial/ethnic disparities [132,133,134].

#### 2.1.2. Study Aims and Hypotheses

Specific aims are to:

1. Identify and compare rates of disruption in cancer care due to the COVID-19 pandemic and health-related quality of life among non-Hispanic Black/African American, Hispanic/Latina, and non-Hispanic White women diagnosed with early-stage breast cancer.
2. Examine how proximal, intermediate, and distant determinants differentially predict cancer patients’ disruption of care and health-related quality of life during and after the acute phase of COVID-19 pandemic.
3. Identify barriers and facilitators of cancer care receipt and health-related quality of life among participants who report high vs. low rates of cancer care disruptions during the COVID-19 pandemic.

It was hypothesized that: (1) differences in cancer care disruption and health-related quality of life will exist among non-Hispanic Black/African American, Hispanic/Latina, and non-Hispanic White women diagnosed with early-stage breast cancer; and that (2) higher rates of disruptions and more affected health-related quality of life will be experienced by participants who identify as non-Hispanic Black/African American or Hispanic/Latina, those with lower socioeconomic status, no insurance, and those reporting greater distress and more negative coping approaches. In addition, it was hypothesized that (3) higher rates of disruptions and more affected quality of life will be experienced by participants with lower social support and reduced social network size. Finally, (4) worse outcomes were expected to be reported by participants receiving care in institutions lacking patient navigation and psychoeducation, access to tele-medicine services, and limited community level resources. 

#### 2.1.3. Recruitment and Study Setting

Community-engaged approaches to survey development and recruitment strategies guide the research project. The investigative team presented the study materials (flyers, scripts and survey instruments) to a local Community Research Advisory Board (see: https://www.healthrcmi.com/crab, accessed on 29 November 2021) and an External Advisory Board (see: https://www.uhandpartnership.com/external-advisory-board, accessed on 29 November 2021), eliciting community-scientists feedback and making modifications accordingly. Participants will be recruited through a variety of strategies to maximize the likelihood of reaching the expected sample size, placing particular emphasis on equal representation among the three groups. Breast cancer survivors will be recruited from local and national community-based organizations and advocacy groups for women’s health and breast cancer prevention, in collaboration with patient advocates and community health workers. In addition, recruitment scripts and flyers will be disseminated via social media, during breast cancer-related community events, and through postings on community bulletin boards in Black/Latinx communities and churches. Study recruitment efforts will be conducted also in target clinic-sites serving women with breast cancer from minority groups, including targeted emails to participants of previous studies who have expressed interest in being contacted for future research. 

#### 2.1.4. Participants

The target sample will comprise 120 breast cancer patients equally divided in groups of 40 non-Hispanic Black/African American, 40 Hispanic/Latina and 40 NHW women. Additionally, purposive sampling will be used to select a subset of 30 women, stratified by race/ethnicity and low vs. high rates of disruption in care, to complete an individual semi-structured interview to further understand factors influencing continuity of care and treatment decision-making. Initially, women will be selected for interviews on a first-come, first-served basis, with attention to ensuring equal representation by race/ethnicity until *n* = 30 is reached. Once data analysis has commenced, theoretical sampling will also guide selection of interviewees in keeping with grounded theory. 

Inclusion criteria for the present study are:

1. Self-identify as non-Hispanic Black/African American, Hispanic/Latina, or NHW woman;
2. Having been diagnosed with early stage (I–III) breast cancer in January 2020 or later;
3. Receiving care for breast cancer at time of enrollment;
4. Being 18 years of age or older;
5. Having access to a computer, smartphone, tablet, or other devices allowing the capability to complete internet-based survey and interview;
6. Having the ability to speak and read English.

The exclusion criteria are:

1. Having a cognitive impairment or severe mental illness;
2. Being unable to consent;
3. Being in prison/custody; or
4. Being diagnosed with metastatic disease.

Potential participants are presented with a list of exclusionary criteria and asked to select out of the study if any inclusion criteria are not met or if any exclusion criteria are applicable.

### 2.2. Data Collection

#### 2.2.1. Quantitative Questionnaire—Measures

*Demographic/medical characteristics*: Participants will be asked sociodemographic information including age, sex at birth, gender identity, race/ethnicity, marital status, duration of current relationship (if applicable), religious affiliation, number of children, occupational status, education, insurance status, income, and residential street address. Medical information will include cancer diagnosis (first diagnosis vs. recurrence), stage of the disease, time since diagnosis, and treatment type.

*Proximal determinants*: Psychological distress is assessed with the Perceived Stress Scale [135,136], consisting of 10 items that examine the degree to which situations in one’s life are appraised as stressful. Coping is measured with the Brief COPE [137], a multidimensional measure assessing 14 coping dimensions. The Brief COPE has been shown to be reliable, valid, and it has been extensively used with breast cancer patients [138,139,140,141]. The Cancer Behavior Inventory-Brief Version (CBI-B) [142], assesses respondents’ self-efficacy in managing the illness. Health literacy is measured with the single health literacy screening measure [143]. Individual risk factors such as smoking, alcohol and substance use, physical activity, and diet (cups of fruits and vegetables consumed) will be assessed with single items [144]. Depression and anxiety will be assessed with the Patient Health Questionnaire-8 [145,146,147] and the GAD-7 [148]. 

*Intermediate determinants*: Social support will be measured with the MOS Social Support Scale [149], a 19-item questionnaire assessing tangible, emotional/informational, affectionate support, and positive social interaction. The PROMIS Social Isolation Scale [150,151,152], 4 items, will assess social isolation. In addition, the Social Network Index (12 items) [153] will examine characteristics of the social network of the individual. Emotional intimacy with a partner will be measured with six items from the Personal Assessment of Intimacy in Relationships (PAIR) Inventory [154]. Local community level resources and characteristics will be examined through GIS data based on geocoding each participant’s residential address [155]. Participants also complete two five-item brief measures of Social Cohesion and Trust and Informal Social Control [156], as well as a 10-item measure of Neighborhood Problems [157].

*Distal determinants*: Participants will complete questions, developed by the investigators, about the healthcare setting where they have been receiving care and will be invited to report whether patient navigation, education and supportive services are offered, and whether these services have transitioned to telehealth because of the pandemic. Additionally, women’s experience with health care services and medical mistrust will be investigated with Short-Form Patient Satisfaction Questionnaire (PSQ-18) [158], and the Group-Based Medical Mistrust Scale [159], a 12-item instrument designed to assess race-based medical mistrust from health care systems/professionals and the treatment provided to individuals of the patient’s ethnic or racial group.

*Disruption in Cancer Care:* Cancer care disruptions will be measured with a series of questions investigating the impact of the COVID-19 outbreak on access to health care services (such as the type of health care services that have been impacted and the reason for the change in care). Questions have been adapted from the ACS CAN COVID-19 Impact on Cancer Patients and Survivors survey with permission [160]. 

*Health-Related Quality of Life:* Health-related quality of life will be assessed with the Functional Assessment of Cancer Therapy-Breast (FACT-B) Scale [161], a 37-item instrument measuring physical, social/family, emotional, and functional well-being, along with breast cancer-specific concerns. In addition, four items from the Behavioral Risk Factor Surveillance System survey [162] have been included. Items assess (a) self-rated health, (b) poor physical health days, (c) poor mental health days, and (d) activity limited days due to poor physical or mental health. Self-rated health is assessed with a single item with which participants rate their health on a 5-point scale from excellent (1) to poor (5). Finally, participants will be invited to report the number of days in the previous 30 days in which poor physical or mental health limited their ability to perform usual activities [162].

#### 2.2.2. Qualitative Interviews

At the end of the online survey, participants will be invited to express their interest in participating in a qualitative interview (*n* = 30). The goal of the interview is to assess factors that influence women’s access to and continuity of care, treatment decision-making, self-reported engagement in the process of care, and overall satisfaction. A semi-structured topic guide will be used to focus interviews; however, it will remain open and flexible to change to respond to participants’ experiences and concerns. In addition, women will be asked about how their social networks facilitated or impaired their cancer treatment management. Those who express interest will be contacted by the research team via e-mail/phone. Members of the research team will share information about the topics covered in the interviews, how interviews will be conducted and how data will be collected. Participants will be orally consented at the beginning of the interview. Instructions will be provided to participants to access the HIPAA—compliant online platform selected for the interview. Online interviews were chosen to reduce the risk of exposure to COVID-19.

### 2.3. Analysis

#### 2.3.1. Sample Size Calculation

Power and sample size requirements were based on (1) Aim 1: one-way analysis of variance (ANOVA) with one between subjects factor (i.e., three racial/ethnic groups), and (2) Aim 2: regression analyses with maximum of eight predictors when examining the effect of differential factors (e.g., proximal, intermediate, distal, and their sub measures). Power analysis showed that there is 80% power to detect a moderate (0.25) to large (0.40) effect size of 0.287 (f) for Aim 1 and 0.067 (f^2^) for Aim 2 [163]. Given one between subjects factor design (Aim 1) and regression analyses (Aim 2), two-sided significant level, an alpha of 0.05, and 120 participants (40 participant in each racial/ethnic group). Additionally, with the sample size of 120, it will be possible to detect as small as w of 0.28 for chi-square tests, and correlation of |r| of 0.25 or larger when assessing the correlation between two continuous variables. 

#### 2.3.2. Data Analysis

Descriptive analyses will be computed to characterize the distributional nature of all variables. ANCOVAs controlling for covariates (e.g., age) will explore mean differences between racial/ethnic groups, whereas chi-square tests will compare participants and those who withdrew on sociodemographic demographic and medical factors. Adjusted means of outcomes of interest for each racial/ethnic group will be reported and pairwise comparisons will be conducted when significant group differences are found. In addition, the association between proximal, intermediate, and distant factors, cancer care disruptions, and health-related quality of life will be examined with bivariate correlations. Then, a series of hierarchical multiple regression analysis will investigate the contribution of the predictors on these outcomes in two steps where variables will be sequentially added and retained: (1) participant characteristics (e.g., age and race/ethnicity); and (2) the addition of proximal, intermediate, or distant factors (step 2). The increase in total explained outcome variance (∆R^2^) will be examined. A final model including significant predictors from hierarchical multiple regression analyses will be examined. To investigate whether the model differs by race, an interaction term of race and the identified significant predictors will be introduced. Post hoc analyses will be conducted if significant interaction effect is found. *p*-values < 0.05 will be considered statistically significant. Statistical analyses will be performed using SAS 9.4 [164]. 

#### 2.3.3. Qualitative Analysis

Interview recordings will be transcribed verbatim. Barriers and facilitators experienced by women who report high vs. low rates of cancer care disruption across the three groups will be assessed using a grounded theory approach [165]. The constant comparison method will be used to distinguish similarities and differences across individual transcripts. Iterative data collection and analysis will be used, such that emerging results will guide subsequent data collection and refining of analytic concepts [166]. Researcher triangulation will ensure rigor in data analysis, in which two or more researchers will independently code transcripts, and meet to discuss any discrepancies in coding until agreement is reached. Transcripts of the semi-structured interviews (*n* = 30) will be analyzed completing three phases of coding to identify the emerging theoretical framework. The first phase, (*open coding*), entails intense line-by-line coding of early data to explore the dimensions and properties of emerging concepts and key processes. Subsequently, these initial codes and concepts will be condensed and organized around a single coding category (*axial coding*) [165]. They will be compared until a condition of theoretical saturation is reached and no new codes are identified. Finally, information will be arranged in a diagram (*selective coding*) synthesizing the emerging theoretical model [165,166,167]. Codes will be inductively derived from the data rather than being determined a priori. Atlas.ti software (ATLAS.ti Scientific Software Development GmbH, Berlin, Germany) will be used to manage and organize qualitative data [168].

## 3. Discussion

Despite growing evidence that supports adverse consequences of COVID-19 on clinical and psychosocial outcomes of cancer patients, a limited number of studies have investigated cancer care disruption and patient-reported outcomes among racial and ethnic minoritized women diagnosed with breast cancer. Given the disproportionate burden of the disease, assessing the impact of the pandemic and associated SDoH among a tri-racial and ethnic sample is critical to inform best-practices for future models of care delivery and to improve care transitions [169,170,171,172]. Specifically, these data will provide the necessary evidence to inform whether a subsequent, multilevel intervention addressing these factors is warranted to improve quality of care delivery during and after the COVID-19 pandemic. The present work will yield information about “at risk” patients that can be used to alert healthcare professionals about patient-level factors to consider when creating plans to improve care transitions. The study will also identify ways to leverage existing social resources (i.e., family caregivers, social network members, community agencies/organizations) to help manage and support patients’ outcomes. 

## 4. Conclusions

This study will add to the cancer health disparities evidence base about the: (1) impact of COVID-19 pandemic on cancer care among non-Hispanic Black/African American and Hispanic/Latina women, relative to non-Hispanic White breast cancer survivors in the U.S., and (2) the differential influence of proximal, intermediate, and distal factors on cancer care receipt and health-related quality of life of women—and specifically vulnerable groups—undergoing treatment for breast cancer during a public health crisis. Ultimately, we seek to mitigate disparities in care and outcomes for women whose health may be disproportionately affected by the effects of the pandemic and its sequelae. 

## Data Availability

Not applicable.
